# Hepatitis B virus infection in a cohort of HIV infected blood donors and AIDS patients in Sichuan, China

**DOI:** 10.1186/1479-5876-12-164

**Published:** 2014-06-12

**Authors:** Yu Liu, Peibin Zeng, Jingxing Wang, Gui Liu, Min Xu, Ling Ke, Miao He, Zhong Liu

**Affiliations:** 1 Experimental center of transfusion medicine, Institute of Blood Transfusion, Chinese Academy of Medical Sciences, Hua Cai Road 26 Hao, Dong San Huan Road Er Duan, Chengdu, Sichuan, China; 2 Department of infectious disease, Institute of Blood Transfusion, Chinese Academy of Medical Sciences, Hua Cai Road 26 Hao, Dong San Huan Road Er Duan, Chengdu, Sichuan, China

**Keywords:** HIV, HBV, Blood donors, AIDS patients

## Abstract

**Background:**

Co-infections of HBV and HIV are frequent due to similar routes of transmission. In that transmission through blood is an important route for both HBV and HIV, evaluation of the prevalence of HBV in HIV infected blood donors may be important for transfusion safety. In addition, because the epidemiological characteristics of HBV in HIV infected patients and blood donors may differ from each other, understanding of it could be significant for therapy and prevention of HBV in HIV infected adults. However, data reported on these in Chinese people remains limited.

**Methods:**

614 HIV confirmed positive samples were collected from blood donors and patients and were screened for HBsAg and HBV DNA. The samples screened reactive for HBsAg or positive for HBV DNA were tested for the other serological markers of HBV including anti-HBs, HBeAg, anti-HBe and anti-HBc. For the samples tested positive for HBV DNA, the S region of HBV was amplified by nested PCR and the HBV genotypes were determined.

**Results:**

HBV coinfections were found in 12.9% (79/614) HIV infected individuals including 42/417(10.1%) blood donors and 37/197 (18.8%) AIDS patients. In the HBsAg positive individuals, 80.0% were HBeAg negative in which 10.0% were HBV DNA negative and 38.3% with HBV DNA lower than 2000 IU/ml. The average HBV DNA levels were lower in donors than in patients. In the HBV DNA positive populations, HBV genotypes B, A and C accounted for 48.1%, 22.8% and 8.86% respectively. Mutations related to the failure of HBsAg detection were found in 2 of the 4 HBsAg-/HBV DNA + subjects.

**Conclusions:**

High prevalence of HBV in HIV infected individuals was found in this study. Hence, we recommend routine testing of HBV for patients newly diagnosed with HIV/AIDS in China. Some HIV-HBV co-infected patients remain undiagnosed if only conventional serological markers for HBV are used and it’s important to detect HBV DNA for HIV infected patients. HBV DNA levels were relatively low in HBeAg negative patients, thus this serologic marker may be useful in prioritizing patients on their need for HBV treatment in settings in which HBV DNA is not available.

## Background

Hepatitis B virus (HBV) and human immunodeficiency virus (HIV) are both major public health problems worldwide. They share similar routes of transmission, namely through blood and blood products, sharing of needles to inject drugs, and sexual activity, resulting in coinfection with the two viruses a common event [1]. Studies reported that coinfection with HBV was a well-documented cause of liver-related complications in individuals with HIV infection and is associated with an increased risk of mortality [2-4]. Because of the significant burden and clinical impact of HBV in HIV-infected individuals, understanding the epidemiologic characteristics of HBV infection in HIV-infected populations is crucial. It has been estimated that HBV accounts for 400 million chronic infections worldwide. The prevalence of HBV infection among HIV-infected persons varies markedly, from 5% to 30% in different regions of the world [5]. HBV infection is highly endemic in China, with 5.84% prevalence of HBV surface antigen (HBsAg) in the population of 1–59 years of age in 2007 [6]. However, data on the prevalence of HBV coinfection in HIV infected adults in China remains limited [7,8].

HBV has ten genotypes (A through J) with wide geographic distribution. Genotypes B and C are most common in Asia, and genotype A, which is more responsive to interferon therapy than are the other genotypes, is most common in Northern and Central Europe [9]. However, the role of HBV genotypes in the natural history of the infection and in the response to therapy in patients with HIV coinfection is unclear. In China, the most common genotypes of HBV in individuals who have HBV infection alone are C and B [10]. However, data on the HBV genotype distribution in HIV/HBV co-infected Chinese individuals still remains limited.

Because of the different clinical characteristics of HIV infected patients and blood donors, the epidemiological characteristics of HBV in the two HIV infected groups may differ from each other, which could be significant for therapy and prevention of HBV in HIV infected adults.

Sichuan is a southwest province in China with a total population of about 89 million. The prevalence of HIV in Sichuan is higher than that in many other provinces and ranks in the top five provinces of highest prevalence in China. Therefore, in this study we investigated the prevalence of HBV in newly diagnosed HIV-infected blood donors and AIDS patients in Sichuan, China and analyzed the HBV genotype distribution, viral loads, serological markers of HBV and their relationships.

## Methods

### Samples

614 HIV-positive samples were collected between 1995 and 2010 which were screened reactive for anti-HIV1/2 by ELISA(enzyme-linked-immunosorbent serologic assay) and then confirmed by western blotting using an HIV 1/2 antibody immunoblot kit (AUSIA, Hangzhou, China) at the HIV Confirmation Laboratory of the Institute of Blood Transfusion, Chinese Academy of Medical Science. The approval from the Ethical Committee of Chinese Academy of Medical Science was gained for the use of these samples for research purposes. All samples collected in different years were from different individuals. Of all the 614 serologically HIV-positive participants, 417 were blood donors (348 male, 69 female) and 197 were clinical patients from hospitals in the province of Sichuan, China (152 male, 45 female) (Table 1). The mean age of these individuals was 34.6 ± 12.3 years (range, 15–79 years). There were 453 (81.4%) men with a mean age of 34.9 ± 12.5 years (range, 15–79 years) and 120 (18.6%) women with a mean age of 33.8 ± 11.7 years (range, 18–67 years). In China, all the blood donors should be screened for anti-HIV by ELISA and the donors screened reactive will be excluded by donation. Thus, all the 417 blood donors were excluded by donation. HBV coinfection was defined as positive for HBsAg or HBV DNA. All the donors and patients were newly diagnosed HIV infected and therapy-naïve individuals.

**Table 1:** Characteristics and HBV coinfection prevalence of HIV infected individuals

	**Total (%)**	**HBV positive (%)**	**Donors**			**Patients**			**P**
	**(N = 614)**	**(N = 79)**	**Total**	**HBV positive**	**%**	**Total**	**HBV positive**	**%**	**Donors VS patients**
			**(N = 417)**	**(N = 42)**		**(N = 197)**	**(N = 37)**		
Total	614	79 (12.9)	417	42	10.1	197	37	18.8	0.003
Gender									
Male	500 (81.4)	66 (13.3)	348	36	10.3	152	30	19.7	0.004
Female	114 (18.6)	13 (11.4)	69	6	8.7	45	7	15.6	0.26
Age									
≤35y	356 (58.0)	46 (12.9)	281	30	8.7	75	16	21.3	0.01
36-45y	127 (20.7)	15 (11.8)	98	8	7.1	29	7	24.1	0.02
>45y	80 (13.0)	8 (10.0)	33	4	11.1	47	4	9.8	0.6
unknown	51 (8.3)	10 (19.6)	5	0	0	46	10	21.7	
Year									
1995-2007	120 (19.5)	16 (13.3)	86	9	10.5	34	7	20.6	0.14
2008	161 (26.2)	22 (13.7)	128	14	10.9	33	8	24.2	0.047
2009	155 (25.2)	21 (13.5)	94	9	9.6	61	12	19.7	0.07
2010	178 (29.0)	20 (11.2)	109	10	9.2	69	10	14.5	0.27

### Serological tests

All the samples were screened for Hepatitis B surface antigen (HBsAg) using ELISA Kits (Kehua Biotechnology, Shanghai, China) according to the manufacturer’s instructions. This kit has not yet been evaluated for performance on HBsAg mutant panels. Confirmatory test (Livzon Diagnostics INC., Zhuhai, China) was conducted on the samples tested reactive for HBsAg by ELISA. For the samples confirmed positive for HBsAg, other four serological markers of HBV were tested including antibody to Hepatitis B surface antigen (anti-HBs), Hepatitis B e antigen (HBeAg), antibody to e antigen (anti-HBe) and antibody to Hepatitis B core antigen (anti-HBc). According to the instructions, the sensitivity of the assay for HBsAg was 0.05 ng/mL.

### Viral DNA extraction and amplification of the S region

Aliquots of plasma (50 μL) from four HIV-positive blood samples were pooled and subjected to nucleic acid extraction using QIAamp DNA Blood Mini Kit (Qiagen, Hilden, Germany) according to the manufacturer’s instructions and 50 μL of eluted DNA was stored at -70°C until use. The HBV S region was amplified by nested PCR using a thermal cycler (Veriti, Applied Biosystems) with BS1 (nt 203–221, 5′-GCGGGGTTTTTCTTGTTGA-3′) as the sense primer and BS2 (nt 788–769, 5′-GGGACTCAAGATGTTGTACAG-3′) and BS3 (nt 712–731, 5′-AAGCCCTACGAACCACTGAA-3′) as the antisense primers for the first-round and second-round PCR, respectively. The first-round PCR was performed with Taq DNA polymerase (Tiangen) in a total volume of 40 μL, with the following reaction variables: predenaturation at 95°C for 5 minutes, followed by 35 cycles of 15 seconds of denaturation (95°C), 30 seconds of annealing (53°C), and 30 seconds of extension (72°C), with a final extension at 72°C for 5 minutes. The cycling conditions of the second-round PCR were the same as the first-round PCR but using 2 μL of the first-round PCR product as template [11]. After nested-PCR, 200 μL of plasma from each sample of HBV DNA positive pools underwent nucleic acid extraction independently using the same protocol as mentioned above and then nested PCR of S region were performed for every extracted DNA. To avoid false positive results, comprehensive procedures were followed to prevent sample cross-contamination, and test results were accepted as valid only when obtained in duplicate.

### DNA sequencing, phylogenetic analysis and multiplex PCR

The PCR products were analyzed by electrophoresis in 1.5% agarose gels and purified using a commercially available kit (NucleoSpin Extract II kit, Macherey-Nagel GmbH & Co. KG, Du¨ren, Germany) according to the manufacturer’s instructions. Purified products were used as templates in cycle sequencing reactions. Nucleotide sequences were determined from both strands using primers BS1 and BS3, and the resulting sequences were read directly with a genetic analyzer (ABI 3730, Applied Biosystems). For the single sequence with no heterogeneous sites, the HBV genotypes were determined by phylogenetic analysis with a panel of reference sequences from genotypes A to H retrieved from GenBank as reported [11]. If the amplified sequence had heterogeneous sites identified by sequencing both strands, multiplex PCR was used for genotyping as reported previously [11].

### Quantitative assay of HBV-DNA

HBV-DNA level of the HBV infected samples, HBsAg positive or HBV DNA detected by nested-PCR, was tested by real-time fluorescence quantitative PCR with TaqMan probe using the Quantitative Hepatitis B Virus PCR Fluorogence Diagnostic Kit (PG Biotechnology, Shenzhen, China) [12]. Standard curves of the reactions were constructed using quantitation standard supplied by manufacturer. The detection limits of this method range from 5 × 10^2^ to 5 × 10^7^ IU/ml using a serum sample volume of 100 ul. The quantitative HBV DNA testing was performed three times on all samples and the average was recorded as the result for the sample.

### Analysis of mutations in the S region

The presence of amino acid mutations in the antigenic loop was analyzed from amino acid 103 to 173 of HBsAg and compared to a consensus sequence from the same genotype. Mutations related to genetic polymorphisms were noted, but the focus was placed on mutations reported to be associated with diagnostic failure [13].

### Statistical analysis

Quantitative data were expressed as mean ± SD and the viral load was expressed as logarithmic transformation of original values. Comparisons between groups were analyzed by chi-square test or Fisher’s exact test for categorical variables and by Student’s t-test for quantitative variables. P-values below 0.05 were considered significant. All statistical analysis was performed using SPSS software for Windows 16.0 (SPSS, Chicago, IL).

## Results

### The prevalence of HBV in HIV infected donors and AIDS patients

Of the 614 HIV infected individuals investigated, 79 (12.9%) were found to have HBV coinfection, including 42 blood donors with mean age of 32.6 ± 8.8 years old and 37 AIDS patients with mean age of 35.1 ± 12.7 years old. The prevalence of HBV coinfection in HIV infected blood donors (42/417, 10.1%) was found significantly lower than that in AIDS patients (37/197, 18.8%, P = 0.003). Of the 79 subjects, 69 were positive for both HBV DNA and HBsAg. There were 6 subjects detected positive for HBsAg but negative for HBV DNA and 4 subjects detected positive for HBV DNA but negative for HBsAg. As shown in Table 1, the prevalence of HBV coinfection in different gender group, different age group and different year group of samples collected was analyzed and no significant difference was found.

### Serological markers of HBV and the HBV DNA levels

**Figure 1:**
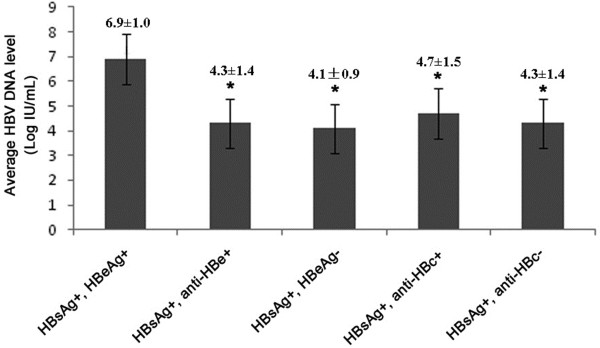
**The average HBV DNA levels in different serological markers groups.** The average HBV DNA level of HBeAg positive subjects was found significantly higher than anti-HBe positive subjects, HBeAg negative subjects, anti-HBc positive subjects and anti-HBc negative subjects (P < 0.001).

As shown in Table 2, of the 75 HBsAg positive subjects, 39 were from HIV infected donors and 36 were from AIDS patients and the average HBV DNA level of the former group was significantly lower than that of the latter group (4.2 ± 1.1 LogIU/ml VS 5.0 ± 1.7LogIU/ml, P = 0.008). Of all the HBsAg positive subjects, 15 (20.0%) were found HBeAg positive and 60 (80.0%) were found HBeAg negative. The rate of HBeAg positivity of HBsAg positive donors (3/39, 7.7%) was significantly lower than that of HBsAg positive patients (12/36, 33.3%, P = 0.01) while the rate of HBeAg negativity of HBsAg positive donors (36/39, 92.3%) was significantly higher than that of HBsAg positive patients (24/36, 66.7%, P = 0.01). In the 60 HBsAg + HBeAg- subjects, 6 (10.0%) were HBV DNA negative and 23 (38.3%) with HBV DNA less than 2000 IU/ml. The average HBV DNA level of HBeAg negative subjects was found significantly lower than HBeAg positive subjects (P < 0.001) (Figure 1). The rate of anti-HBc positivity in HBsAg positive donors (28/39, 71.8%) was significantly lower than that in HBsAg positive patients (34/36, 94.4%, P = 0.009). As shown in Table 3, of the 13 HBsAg carriers negative for anti-HBc, 11 were negative for both HBeAg and anti-HBe and 2 were HBeAg positive. 11 of the 13 samples tested positive for HBV DNA. All the HBsAg reactivity was confirmed by neutralization test.

**Table 2:** Serological markers of HBV and the HBV DNA levels

**HBV serological status**	**Number tested (n = 79)**	**%**	**P (% of donors VS% of patients)**	**HBV DNA level (Log IU/mL)**	**P (HBV DNA level of donors VS patients)**
HBsAg^+^	75	94.9(75/79)		4.6 ± 1.5	
Donors	39	92.9(39/42)		4.2 ± 1.1	0.008
Patients	36	97.3(36/37)		5.0 ± 1.7	
HBsAg+ ,HBeAg+	15	20.0(15/75)		6.9 ± 1.0	
Donors	3	7.7(3/39)	0.01	6.6 ± 0.5	0.24
Patients	12	33.3(12/36)		6.9 ± 1.1	
HBsAg+ ,HBeAg-	60	80.0(60/75)		4.1 ± 0.9	
Donors	36	92.3(36/39)	0.01	4.1 ± 0.9	0.75
Patients	24	66.7(24/36)		4.1 ± 0.9	
HBsAg+ ,anti-HBe+	19	25.3(19/75)		4.3 ± 1.4	
Donors	7	17.9(7/39)	0.13	3.9 ± 0.7	0.12
Patients	12	33.3(12/36)		4.6 ± 1.6	
HBsAg+ ,anti-HBc+	62	82.7(62/75)		4.7 ± 1.5	
Donors	28	71.8(28/39)	0.009	4.7 ± 1.3	0.41
Patients	34	94.4(34/36)		4.8 ± 1.6	
HBsAg+,anti-HBc-	13	17.3(13/75)		4.3 ± 1.4	
Donors	11	28.2(11/39)	0.009	3.7 ± 0.6	0.006
Patients	2	5.56(2/36)		6.6 ± 1.5	

**Table 3:** The information of the HBsAg+/anti-HBc- subjects*

**Samples**	**Gender**	**Age**	**Source**	**Anti-HBs**	**HBeAg**	**Anti-HBe**	**HBV DNA**
1	Female	39	Patient	N	P	N	P
2	Female	41	Donor	N	N	N	P
3	Female	31	Donor	P	N	N	P
4	Female	49	Donor	N	N	N	P
5	Female	26	Donor	N	N	N	P
6	Male	25	Patient	N	P	N	P
7	Male	54	Donor	N	N	N	P
8	Male	24	Donor	P	N	N	N
9	Male	25	Donor	P	N	N	P
10	Male	27	Donor	P	N	N	P
11	Male	30	Donor	P	N	N	P
12	Male	34	Donor	P	N	N	N
13	Male	18	Donor	N	N	N	P

P: positive; N: negative.

*:All the samples were confirmed by neutralization.

### HBV genotypes in the HIV infected donors and AIDS patients

**Figure 2:**
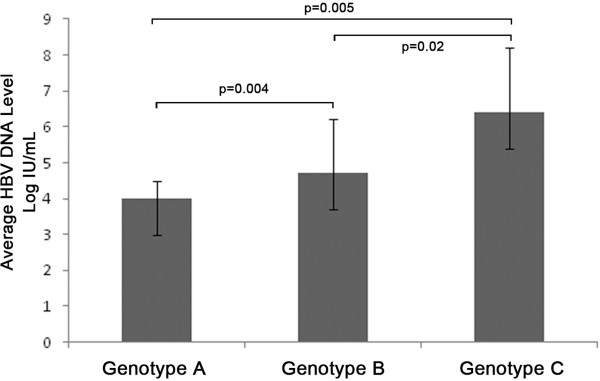
**The average HBV DNA levels of different HBV genotype groups.** The average HBV DNA level of the subjects infected with genotype A HBV was significantly lower than that of the subjects infected with genotype B (P = 0.004) and genotype C HBV (P = 0.005). The average HBV DNA level of the subjects infected with genotype B HBV was found significantly lower than that of the subjects infected with genotype C HBV (P = 0.02).

A total of 67 (91.8%) of the 73 HBV DNA positive samples were successfully genotyped, of which 18 (22.8%, 95%CI: 13.5%-32.0%) were found infected with genotype A HBV, 38 (48.1%, 95%CI: 37.1%-59.1%) with genotype B, 7 (8.86%, 95%CI: 2.59%-15.1%) with genotype C, 3 (3.80%, 95%CI: 0.42%-8.01%) with genotype D, 1 (1.27%, 95%CI: 0.20%-3.73%)with mixture of genotype B and D and no subjects with genotypes E to H. No significant difference was found in the genotypes distribution of the two groups. The average HBV DNA level of the subjects infected with genotype A HBV was 4.0 ± 0.5 Log IU/mL which was significantly lower than that of the subjects infected with genotype B (4.7 ± 1.5 Log IU/mL, P = 0.004) and genotype C HBV (6.4 ± 1.8 Log IU/mL, P = 0.005, Figure 2). In addition, the average HBV DNA level of the subjects infected with genotype B HBV was found significantly lower than that of the subjects infected with genotype C HBV (P = 0.02, Figure 2).

### The characteristics of the HBsAg negative HIV/HBV coinfection subjects

Of the 79 HBV infected subjects, 4 (0.65%, 4/614) were found HBsAg negative including 3 donors (0.72%, 3/417) and 1 patient (0.51%, 1/197). The information of the 4 subjects was listed in Table 4. All the four subjects were younger than 40 years old with one female (number 1) and 3 males (number 2 to 4). For number1, the HBV DNA level was 1.44 × 10^5^ which was higher than the viral load of number 2 to 4. The HBeAg and anti-HBe were tested positive for number 1 while negative for all the other three males. All the four subjects were tested positive for anti-HBc. The subject of number 1 was found infected with genotype C HBV while number 3 and 4 infected with genotype B HBV and number 2 infected with HBV of undetermined genotype. In addition, the sequence analysis showed that mutations of P127S and G145R occurred in the HBV infected by number 1 subject and mutation of L109M occurred in number 3 infected HBV while no mutation was found in the HBV infected by number 2 and 4.

**Table 4:** The information of the HBsAg-/HBV DNA + subjects*

**Samples**	**Gender**	**Age**	**Source**	**Collection year**	**HBV DNA Level (IU/ml)**	**HBV Genotype**	**HBeAg**	**anti-HBe**	**anti-HBs**	**Mutations**
1	Female	21	Donor	2008	1.44 × 10^5^	C	P	P	N	P127S and G145R
2	Male	35	Donor	2009	<500	ID	N	N	N	NO
3	Male	23	Donor	2010	1.08 × 10^3^	B	N	N	P	L109M
4	Male	38	Patient	2007	3.35 × 10^3^	B	N	N	N	NO

ID: indeterminate; P: positive; N: negative.

*:All the four subjects were positive for anti-HBc.

## Discussion

Coinfection of HBV with HIV complicates the clinical course, management and may also adversely affect therapy for HIV infection. Treatment of patients with HIV/AIDS coinfected with HBV is even more complex [14,15]. Thus it is important to determine whether patients with HIV/AIDS are coinfected with HBV. All over the world, increasing attention has focused on HIV coinfection with HBV in recent years [5] and several studies have reported the HIV/HBV prevalence in China [7,8]. However, most of the studies just adopted seroepidemiological survey methods to detect HBsAg to initially assess the status of HBV infections in HIV/AIDS populations in China, which may underestimate the actual prevalence of HBV for many reasons including window period infection, HBsAg detecting negative for mutations in S region and occult HBV infection. In this study we conducted both HBsAg and HBV DNA detection to assess the HBV prevalence in HIV infected populations and got a result of 12.9% which was higher than the HBV prevalence in the Chinese general population [6]. In addition, the data we got suggests higher HIV/HBV prevalence in Chinese populations than reported by other studies [7,8]. One reason may be that HBV DNA detection was conducted and some HBsAg negative HBV DNA positive subjects were found in this study. In addition, HBV and HIV sole prevalence in the general population in different regions may affect the prevalence of HIV/HBV coinfection determined in different researches and both HBV and HIV are more endemic in Sichuan, the region we studied, than many other provinces in China. The HBV/HIV coinfection prevalence of 12.9% in the present study is significant and confirms that HBV is a major threat to HIV infected individuals in China, as reported in other parts of the world [5]. In our study, the HBV coinfection prevalence was determined separately in HIV infected blood donors and AIDS patients and the result showed that the HBV prevalence in the former group was significantly lower than that in the latter group, which may be due to that the HBV prevalence in Chinese blood donors was lower than that in the general Chinese people.

In this study, more HBeAg negative individuals were found in the HBsAg positive donors than in the patients and the average HBV DNA levels were determined lower in donors than in patients, which may explain why most of the HBV/HIV infected blood donors are asymptomatic. In addition, in this study, 10% of the HBeAg-negative patents had an undetectable HBV DNA, and an additional 38.3% had a HBV DNA level less than 2000 IU/ml, which is the level above which treatment for HBV infection is considered. In contrast, all of the HBeAg-positive subjects had detectable HBV DNA levels that were more than 10^5^ IU/ml. In the Nigerian study, these low levels were also found primarily in HBeAg-negative patients and these findings are supported by the multivariable analysis by other studies demonstrating that HBeAg-positive status was the only factor associated with higher HBV DNA levels [16]. Thus, it is possible to detect HBeAg to determine if HBV treatment needs to be started in HIV infected individuals when HBV DNA assays are not available in resource-limited settings.

Genotypes C and B were the major HBV genotypes endemic in Mainland China in the individuals with HBV infection alone and genotype C is more prevalent than genotype B in general populations in Sichuan province [10]. In this study, we found the most prevalent genotype was B and followed by genotype A and C in the HBV/HIV coinfected individuals, which was quite different from the genotype distribution in the population with HBV infection alone. This may explain the high rate of HBeAg negative individuals in the HBV/HIV coinfected populations detected in this study in that genotype B HBV is less virulent than genotype C and genotype B has a higher rate of seroconversion from HBeAg to anti-HBe. If the HBV genotype distribution demonstrated in our study is the fact, it’s good news for HBV/HIV coinfected populations. However, there is still one question. It is known that HIV infection would impair the cell mediated responses and enhances the kinetics of HBV replication, thus, genotype C HBV, which has greater capability to replicate, is considered to be more likely to infect the HIV infected individuals. This seems to be contradictive with HBV genotype distribution determined in our study. Thus, more studies are needed to determine the HBV genotype distribution in the HBV/HIV coinfected individuals and its mechanism needs to be analyzed in the future.

Through HBV DNA detection, 6 HBsAg positive but HBV DNA negative subjects were found in our study. It seems to be very rare to find HBsAg in the absence of HBV DNA which may be due to high sensitivity of HBV DNA test by nested-PCR used in this study. However, the existence of HBsAg+/HBV DNA- samples suggested that the HBV DNA testing can’t substitute the testing of HBsAg in blood donation screening. In addition, 4 HBV DNA positive but HBsAg negative subjects were found. A number of explanations for the positivity of HBV DNA in HBsAg negative samples have been proposed, including window period infection, occult HBV infection (OBI), genetic variations in the S gene and the presence of immune complexes in which HBsAg may be hidden [17]. To analyze the cause of HBsAg negative, we analyzed the sequence of S gene of the four samples and found three mutations in two subjects. All of the three mutations were located in the sequence coding the central major hydrophilic region (MHR) from residues 103–173 which was exposed at the surface of viral particles and may be related to the failure of HBsAg detection. In the 4 subjects, subject 1 was found with G145R mutation, which was reported as the most important and best-documented mutation in MHR and was reported as the most critical substitution to prevent HBsAg detection. In addition, subject 1 was found positive for HBeAg and with high viral load. Thus, Sample 1, very much likely, was a failure in HBsAg detection due to the outdated quality of the method used, which manufacturer has not yet taken care to assess its performance for detecting HBsAg mutants. It’s perfectly known at present that appropriate modifications in assay design improve such performance if implemented, including a significant improvement of the ability to detect samples containing G145R mutants [18,19]. Therefore, the methods used for testing HBsAg should be carefully selected for avoiding such diagnostic mistakes. Occult HBV infection (OBI) is characterized by positive HBV-DNA in serum (and/or hepatic tissues) of individuals negative for serum HBsAg. A study in Germany showed that the occult HBV infection rate in patients with HIV/AIDS was 2.9% [20]. Although occult hepatitis B infection alone may not have clinical consequences, it may become injurious when the virus is reactivated after immunosuppression, which suggests that HIV coinfection is a high risk for OBI [21,22]. According to the serological markers testing results and HBV DNA load, subject 3 can be determined as OBI. Subjects 2 and 4 were detected positive for only anti-HBc but not for the other four serological markers. Thus, it’s difficult to determine them as OBI unless anti-HBc IgM testing would be done to exclude that they were during the seroconversion phase of the acute HBV infection.

The current study had some limitations. Firstly, the sample size was relatively small and it was a single time point testing without any follow-up. Secondly, Since it was a cross-sectional study, liver disease characteristics such as alanine aminotransferase (ALT)/aspartate aminotransferase (AST) levels and HIV disease characteristics including CD4+ T-cell counts and HIV RNA levels were not determined in this study and we could not comment on the clinical significance of HBV in HIV infected populations included in our study. Thirdly, the data of HIV acquired route of the subjects were not collected, thus it’s impossible for us to analyze the impact of HIV transmission mode on HBV coinfection. Lastly, a control group of HIV negative was not included in the study and we could not compare the HBV infection in the HIV positive group with the negative group. However, some data about the HBV infection in general Chinese people were used to do the comparison.

## Conclusions

The identification of a high prevalence of HBV in HIV infected individuals in this study indicates the significance of screening for HBV among patients newly diagnosed with HIV/AIDS. Hence, we strongly recommend that routine testing for patients newly diagnosed with HIV/AIDS should include tests for HBV in China and that treatment of HBV should become an important part of HIV care. The hepatitis B infection with negative HBsAg in HIV infected Chinese individuals found in this study suggested that HIV/HBV coinfected patients remain undiagnosed, if only conventional serological markers for HBV are used and it’s important to detect HBV DNA for HIV infected patients. Reagents with the ability to detect samples containing mutants should be selected for HBsAg testing to avoid diagnostic mistakes resulting from mutations in MHR region. HBV DNA levels were relatively low in HBeAg negative patients, thus this serologic marker may be useful in prioritizing patients on their need for HBV treatment in settings in which HBV DNA is not available. More studies are needed to determine the HBV genotype distribution in the HBV/HIV coinfected individuals further.

## Abbreviations

HBV: Hepatitis B virus; HIV: Human immunodeficiency virus; ELISA: Enzyme-linked-immunosorbent serologic assay.

## Competing interests

The authors declare that they have no competing interests.

## Authors’ contributions

MH and ZL were responsible for conceptualizing the study. JW critically reviewed and revised the manuscript. YL and PZ analyzed the data and drafted the manuscript. The other authors were responsible for the detection of the samples. All the authors read and approved the manuscript.
